# A unified approach for debugging is-a structure and mappings in networked taxonomies

**DOI:** 10.1186/2041-1480-4-10

**Published:** 2013-03-31

**Authors:** Patrick Lambrix, Valentina Ivanova

**Affiliations:** 1 Department of Computer and Information Science / Swedish e-Science Research Centre, Linköping University, 581 83 Linköping, Sweden

## Abstract

**Background:**

With the increased use of ontologies and ontology mappings in semantically-enabled applications such as ontology-based search and data integration, the issue of detecting and repairing defects in ontologies and ontology mappings has become increasingly important. These defects can lead to wrong or incomplete results for the applications.

**Results:**

We propose a unified framework for debugging the is-a structure of and mappings between taxonomies, the most used kind of ontologies. We present theory and algorithms as well as an implemented system RepOSE, that supports a domain expert in detecting and repairing missing and wrong is-a relations and mappings. We also discuss two experiments performed by domain experts: an experiment on the Anatomy ontologies from the Ontology Alignment Evaluation Initiative, and a debugging session for the Swedish National Food Agency.

**Conclusions:**

Semantically-enabled applications need high quality ontologies and ontology mappings. One key aspect is the detection and removal of defects in the ontologies and ontology mappings. Our system RepOSE provides an environment that supports domain experts to deal with this issue. We have shown the usefulness of the approach in two experiments by detecting and repairing circa 200 and 30 defects, respectively.

## Background

In recent years many biomedical ontologies (e.g., [1]) have been developed. Intuitively, ontologies can be seen as defining the basic terms and relations of a domain of interest, as well as the rules for combining these terms and relations [2]. They are a key technology for the Semantic Web. The benefits of using ontologies include reuse, sharing and portability of knowledge across platforms, and improved documentation, maintenance, and reliability. Ontologies lead to a better understanding of a field and to more effective and efficient handling of information in that field. The work on ontologies is recognized as essential in some of the grand challenges of genomics research [3] and there is much international research cooperation for the development of ontologies.

Often we would want to be able to use multiple ontologies. For instance, companies may want to use community standard ontologies and use them together with company-specific ontologies. Applications may need to use ontologies from different areas or from different views on one area. Ontology builders may want to use already existing ontologies as the basis for the creation of new ontologies by extending the existing ontologies or by combining knowledge from different smaller ontologies. In each of these cases it is important to know the relationships between the terms in the different ontologies. Further, the data in different data sources in the same domain may have been annotated with different but similar ontologies. Knowledge of the inter-ontology relationships would in this case lead to improvements in search, integration and analysis of data. It has been realized that this is a major issue and much research has recently been done on ontology alignment, i.e., finding mappings between terms in different ontologies (e.g., [4]).

Neither developing ontologies nor aligning ontologies are easy tasks, and often the resulting ontologies and alignments are not consistent or complete. Such ontologies and alignments, although often useful, also lead to problems when used in semantically-enabled applications. Wrong conclusions may be derived or valid conclusions may be missed. Defects in ontologies can take different forms (e.g., [5]). Syntactic defects are usually easy to find and to resolve. Defects regarding style include such things as unintended redundancy. More interesting and severe defects are the modeling defects which require domain knowledge to detect and resolve, and semantic defects such as unsatisfiable concepts and inconsistent ontologies. Most work up to date has focused on debugging (i.e., detecting and repairing) the semantic defects in an ontology (e.g., [5-8]). Modeling defects have been discussed in [9-11]. Recent work has also started looking at repairing semantic defects in a set of mapped ontologies [11,12] or the mappings between ontologies themselves [13-15]. In this paper we tackle the problems of debugging the is-a structure of a fundamental kind of ontologies, i.e., taxonomies, as well as the debugging of the mappings between taxonomies in a unified approach.

In addition to its importance for the correct modeling of a domain, the structural information in ontologies is also important in semantically-enabled applications. For instance, the is-a structure is used in ontology-based search and annotation. In ontology-based search, queries are refined and expanded by moving up and down the hierarchy of concepts. Incomplete and wrong structure in ontologies influences the quality of the search results. As an example, suppose we want to find articles in the MeSH (Medical Subject Headings [16], controlled vocabulary of the National Library of Medicine, US) Database of PubMed [17] using the term *Scleral Diseases* in MeSH. By default the query will follow the hierarchy of MeSH and include more specific terms for searching, such as *Scleritis*. If the relation between *Scleral Diseases* and *Scleritis* is missing in MeSH, we will miss 738 articles in the search result, which is about 55% of the original result set. The structural information is also important in ontology engineering research. For instance, most current ontology alignment systems use structure-based strategies to find mappings between the terms in different ontologies (e.g., overview in [18]) and the modeling defects in the structure of the ontologies have an important influence on the quality of the ontology alignment results [19]. Also incomplete alignments and wrong mappings lead to problems for semantically-enabled applications. For instance, the lack of a mapping can lead to the fact that information about similar entities in different databases cannot be integrated. Wrong mappings will lead to wrong results in data integration.

As the ontologies grow in size, it is difficult to ensure the correctness and completeness of the structure of the ontologies. Some structural relations may be missing or some existing or derivable relations may be unintended. This is not an uncommon case. It is well known that people who are not expert in knowledge representation often misuse and confuse equivalence, is-a and part-of (e.g., [20]), which leads to problems in the structure of the ontologies. For instance, we have shown in [21] that many is-a relations were missing in the 2008 and 2009 versions of the ontologies Adult Mouse Anatomy Dictionary (AMA, [22]) and the NCI Thesaurus anatomy (NCI-A, [23]) in the Anatomy track of the Ontology Alignment Evaluation Initiative (OAEI, [24]). Further, it is also difficult to ensure the correctness and completeness for the mappings. Often, the mappings are generated by ontology alignment systems. The system that performed best in the 2011 version of the OAEI Anatomy track had a precision of 0.943 (i.e., there are wrong mappings), a recall of 0.892 (i.e., the alignment is incomplete) and a recall+ (recall for non-trivial cases) of 0.728. The organizers also state that less than half of the participating systems produced good or acceptable results [25].

Semantically-enabled applications require high-quality ontologies and mappings. A key step towards this is debugging the ontologies and their alignments. In this paper we deal with taxonomies connected via mappings in an ontology network. We propose a semi-automatic approach which helps a domain expert to debug the is-a structure of the ontologies and the mappings between them. This includes the detection of defects as well as the repairing of defects. An advantage of our approach is that no external knowledge is needed and, as we will see, much debugging can already be done in this way. However, in the case external knowledge is available, this can be used as well. For instance, in our work we use external resources, such as WordNet [26] and UMLS [27], to recommend user actions. We also note that, although we focus on ontologies in a network, the repairing methods can also be used for single ontologies when wrong and missing is-a relations are available.

The remainder of this paper is organized as follows. In Section ‘Methods’ we present the setting, an overview of our approach, the algorithms and our implemented system. Further, we present experiments in Section ‘Experiments’ and a discussion and related work in Section ‘Discussion and related work’. The paper concludes in Section ‘Conclusions’. For formal definitions of the presented notions and proofs of statements, we refer to the Additional file 1.

## Methods

### Preliminaries

The **ontologies **that we study are **taxonomies**, which are defined using named concepts and subsumption axioms (is-a relations between concepts). Most ontologies contain such axioms and many of the most well-known and used ontologies in the life sciences are covered by this setting. The ontologies are connected into a **network **through **alignments **which are sets of **mappings**^a ^between concepts from two different ontologies. We currently consider mappings of the type *equivalent *(≡), *subsumed-by *(→) and *subsumes *(←). The concepts that participate in mappings we call **mapped concepts**. Concepts can participate in multiple mappings. The domain knowledge of an ontology network is represented by its **induced ontology**. The induced ontology for an ontology network is an ontology, whose concepts are the concepts of the ontologies in the network, and its set of asserted is-a relations contains the asserted is-a relations of the ontologies together with the is-a relations representing the mappings in the alignments in the network.

**Figure 1:**
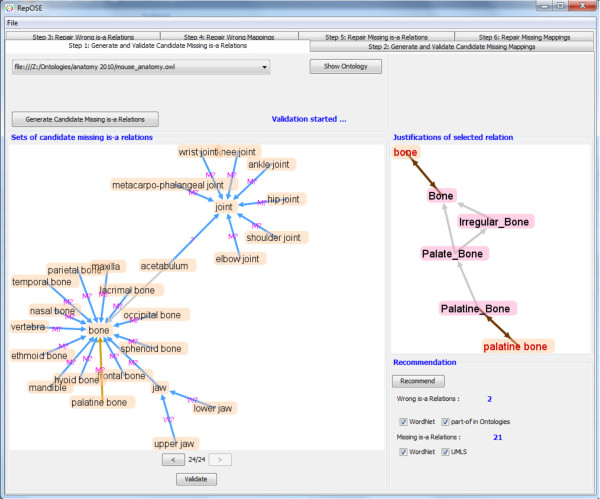
**(Part of an) Ontology network. **Example of an ontology network with two ontologies (with roots ‘bone’) and an alignment.

Figure 1 shows a small ontology network with two ontologies (concepts are represented by nodes and the is-a structures are represented by directed edges) and an alignment (represented by dashed edges).^b ^The alignment consists of 7 equivalence mappings. One of these mappings represents the fact that the concept *bone *in the first ontology is equivalent to the concept *bone* in the second ontology. As these two concepts appear in a mapping, they are mapped concepts.

### Debugging workflow

**Figure 2:**
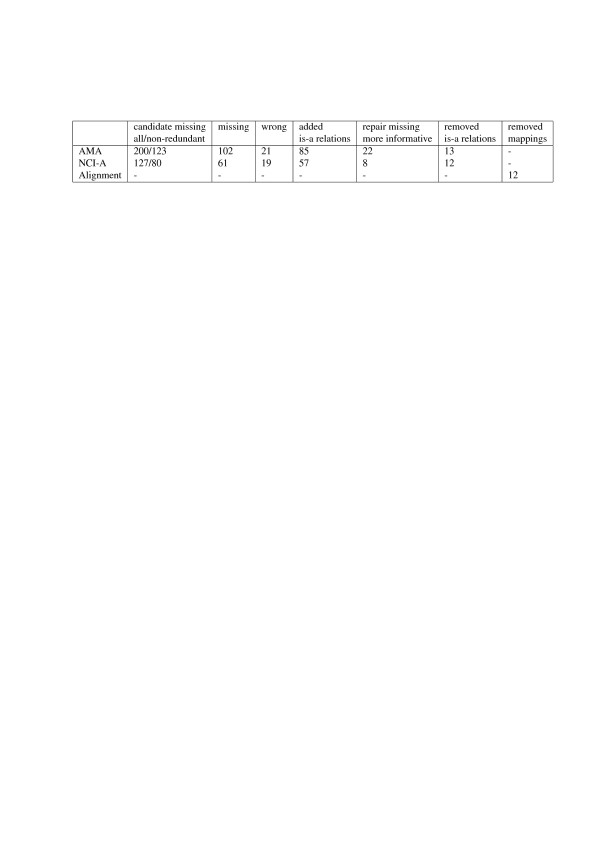
**Debugging workflow. **Overview of debugging workflow with different phases.

In this Subsection, we give an overview of our debugging approach. As illustrated in Figure 2, the process consists of 6 phases, where the first two phases are for the detection and validation of possible defects, and the last four are for the repairing. The input is a network of ontologies. The output is the set of repaired ontologies and alignments.

As discussed before, in this paper we focus on detecting wrong and missing is-a relations and mappings in the ontology network, based on knowledge that is *intrinsic *to the network. Therefore, given an ontology network, we use the domain knowledge represented by the ontology network to detect the deduced is-a relations in the network. For each ontology in the network, the set of **candidate missing is-a relations derivable from the ontology network **(CMIs) consists then of is-a relations between two concepts of the ontology, which can be inferred using logical derivation from the induced ontology of the network, but not from the ontology alone. Similarly, for each pair of ontologies in the network, the set of **candidate missing mappings derivable from the ontology network **(CMMs) consists of mappings between concepts in the two ontologies, which can be inferred using logical derivation from the induced ontology of the network, but not from the two ontologies and their alignment alone. Therefore, one way to start the debugging process is to choose an ontology in the network and in **Phase 1 **CMIs are detected in this ontology. Another way to start the process is to select a pair of ontologies in the network and their alignment. In this case in **Phase 1 **CMMs are detected.

Since the structure of the ontologies may contain wrong is-a relations and the alignments may contain wrong mappings, some of the CMIs and CMMs may be derived due to some wrong is-a relations and mappings. Therefore, we need to validate the CMIs for all ontologies and partition them into **missing is-a relations **and **wrong is-a relations**. Similarly, the CMMs are validated and partitioned into **missing mappings **and **wrong mappings**. This validation (possibly based on recommendations from the debugging system) should be done by a domain expert and is performed in **Phase 2 **of the debugging process. We note that each validation leads to a debugging opportunity. In the case of a wrong is-a relation or mapping, some is-a relations and/or mappings need to be removed. In the case of a missing is-a relation or mapping, some is-a relations and/or mappings need to be added. This is a consequence and an advantage of our logic-based approach using the knowledge intrinsic to the network. The algorithm for detecting the CMIs and CMMs and the procedure for validating are explained in Subsection ‘Detecting and validating candidate missing is-a relations and mappings’.

Once missing and wrong is-a relations and mappings have been obtained, we need to repair them (**Phase 3**).^c ^For each ontology in the network, we want to repair the is-a structure in such a way that (i) the missing is-a relations can be derived from their repaired host ontologies and (ii) the wrong is-a relations can no longer be derived from the repaired ontology network. In addition, for each pair of ontologies, we want to repair the mappings in such a way that (iii) the missing mappings can be derived from the repaired host ontologies of their mapped concepts and the repaired alignment between the host ontologies of the mapped concepts and (iv) the wrong mappings can no longer be derived from the repaired ontology network. To satisfy requirement (i), we need to add a set of is-a relations to the host ontology. To satisfy requirement (iii), we need to add a set of is-a relations to the host ontologies of the mapped concepts and/or mappings to the alignment between the host ontologies of the mapped concepts. To satisfy requirements (ii) and (iv), a set of asserted is-a relations and/or mappings should be removed from the ontology network. The notion of **structural repair **formalizes this. It contains is-a relations and mappings that should be added to or removed from the ontologies and alignments to satisfy these requirements. These is-a relations and mappings are called **repairing actions**.

As explained in our previous work [10] regarding missing is-a relations, not all structural repairs are equally useful or interesting for a domain expert. Therefore, we defined several heuristics of which we use extensions in this work. The first heuristic (pref1) states that we want to use repairing actions that contribute to the repairing. Secondly, we want to repair with as informative as possible repairing actions (pref2). As an example, consider the missing is-a relation *(nasal bone, bone) *in the first ontology in Figure 1. Knowing that *nasal bone *→ *viscerocranium bone*, according to the definition of more informative (see Additional file 1), we know that *(viscerocranium bone, bone) *is more informative than *(nasal bone, bone)*. As *viscerocranium bone *actually is a sub-concept of *bone *according to the domain, a domain expert would prefer to use the more informative repairing action for the given missing is-a relation.^d ^The third heuristic (pref3) prefers not to introduce equivalence relations between concepts A and B when in the original ontology there is an is-a relation betwen A and B. Finally, the single relation heuristic (pref4) assumes that it is more likely that the ontology developers have missed to add single is-a relations, rather than chains of is-a relations.

A naive way of repairing would be to compute all possible structural repairs for the network with respect to the validated missing is-a relations and mappings for all the ontologies in the network. This is infeasible in practice as it involves all the ontologies and alignments and all the missing and wrong is-a relations and mappings in the network. It is also hard for domain experts to choose between structural repairs containing large sets of repairing actions for all the ontologies and alignments. Therefore, in our approach, we repair ontologies and alignments one at a time.

For the selected ontology (for repairing is-a relations) or for the selected alignment and its pair of ontologies (for repairing mappings), a user can choose to repair the missing or the wrong is-a relations/mappings **(Phase 3.1-3.4)**. Although the algorithms for repairing are different for missing and wrong is-a relations/mappings, the repairing goes through the phases of generation of repairing actions, the ranking of is-a relations/mappings, the recommendation of repairing actions and finally, the execution of repairing actions. In **Phase 3.1 **repairing actions are generated. For missing is-a relations and mappings these are is-a relations and/or mappings to add, while for wrong is-a relations and mappings, these are is-a relations and/or mappings to remove. In general, there will be many is-a relations/mappings that need to be repaired and some of them may be easier to start with such as the ones with fewer repairing actions. We therefore rank them with respect to the number of possible repairing actions **(Phase 3.2)**. After this, the user can select an is-a relation/mapping to repair and choose among possible repairing actions. To facilitate this process, we developed methods to recommend repairing actions **(Phase 3.3)**. Once the user decides on repairing actions, the chosen repairing actions are then removed (for wrong is-a relations/mappings) from or added (for missing is-a relations/mappings) to the relevant ontologies and alignments and the consequences are computed **(Phase 3.4)**. For instance, by repairing one is-a relation/mapping some other missing or wrong is-a relations/mappings may also be repaired or their repairing actions may change. Further, new CMIs and CMMs may be found. A description of the algorithms and components of our implemented system RepOSE for **Phases 3.1-3.4 **are found in Subsection ‘Repairing wrong is-a relations and mappings’ (related to wrong is-a relations/mappings) and Subsection ‘Repairing missing is-a relations and mappings’ (related to missing is-a relations/mappings).

We also note that at any time during the process, the user can switch between different ontologies, start earlier phases, or switch between the repairing of wrong is-a relations, the repairing of missing is-a relations, the repairing of wrong mappings and the repairing of missing mappings. The process ends when there are no more CMIs, CMMs, missing or wrong is-a relations and mappings to deal with.

### Algorithms

#### Detecting and validating candidate missing is-a relations and mappings

The CMIs and CMMs could be found using a brute-force method by checking each pair of concepts in the network. If the concepts in a pair belong to the same ontology, and an is-a relation is not derivable from the ontology but derivable from the network, then it is a CMI. If the concepts in the pair belong to different ontologies, and a mapping is derivable from the network, but not from the host ontologies of the concepts and their alignment, then it is a CMM. However, for large ontologies or ontology networks, this is infeasible. Further, some of these CMIs and CMMs are redundant in the sense that they can be repaired by the repairing of other CMIs and CMMs. Therefore, instead of checking all pairs of concepts in the network we define a subset of the set of all pairs of concepts in the network that we will consider for generating CMIs and CMMs. This subset will initially consist of all pairs of mapped concepts. The reason for this choice is that, in the restricted setting where we assume that all existing is-a relations in the ontologies and all existing mappings in the alignments are correct (and thus the debugging problem does not need to consider wrong is-a relations and mappings), it can be shown that all CMIs and CMMs will be repaired when we repair the CMIs and CMMs between *mapped concepts *(see Additional file 1). This guarantees that for the part of the network for which the is-a structure and mappings are correct, we find all CMIs and CMMs when using the set of all pairs of mapped concepts. In addition, we may generate CMIs that were derived using wrong information. These may later be validated as correct or wrong. As our debugging approach is iterative, after repairing, larger and larger parts of the network will contain only correct is-a structure and mappings. When finally all the network contains only correct is-a structure and mappings, it is guaranteed that all defects that can be found using the knowledge intrinsic to the network, are found using our approach.

Therefore, our detection algorithm is applied to mapped concepts^e^. First, we initialize a knowledge base^f^ for the ontology network (*KB*_*N*_). Then, we initialize for each ontology a knowledge base. Further, we initialize a knowledge base for each pair of ontologies and their alignment. For each pair of mapped concepts within the same ontology, we check whether an is-a relation between the pair can be derived from the knowledge base of the network, but not from the knowledge base of the ontology, and if so, it is a CMI. Similarly, for each pair of mapped concepts belonging to two different ontologies, we check whether an is-a relation between the pair can be derived from the knowledge base of the network, but not from the knowledge base of the two ontologies and their alignment, and if so, it is a CMM.

Among these generated CMIs and CMMs, we also remove redundant ones. The remaining CMIs and CMMs should then be validated. As noted before, every CMI or CMM that is generated by our approach gives an opportunity for debugging. If a CMI or CMM is validated to be correct, then information is missing and is-a relations or mappings need to be added; otherwise, some existing information is wrong and is-a relations and/or mappings need to be removed. After repairing, new CMIs and CMMs may be generated.

#### Initialization of the repairing phase

1. Initialize *KB*_*N *_with ontology network N; 

2. For k := 1.. n: 

 initialize *KB*_*k *_with ontology $O_{k}$; 

3. For i := 1.. n-1: for j := i+1.. n: 

 initialize *KB*_*ij *_with ontologies $O_{i}$ and $O_{j}$; 

 for every mapping $(m,n)\in M_{\text{ij}}$: add the axiom *m *→ *n *to *KB*_*ij*_; 

4. For k:= 1.. n: 

 for every missing is-a relation $(a,b)\in {MI}_{k}$: 

 add the axiom *a *→ *b *to *KB*_*N*_; 

 add the axiom *a *→ *b *to *KB*_*k*_; 

 for i := 1.. k-1: 

 add the axiom *a*→*b* to *KB*_*ik*_; 

 for i := k+1.. n: 

 add the axiom *a*→*b* to *KB*_*ki*_; 

5. For i := 1.. n-1: for j := i+1.. n: 

 for every missing mapping $(m,n)\in {MM}_{\text{ij}}$: 

 add the axiom *m *→ *n *to *KB*_*N*_; 

 add the axiom *m *→ *n *to *KB*_*ij*_; 

6.

$$MI:={MI}_{N}$$;

$WI:={WI}_{N}$; $MM:={MM}_{N}$; $WM:={WM}_{N}$; 

7.

$$R_{I}^{+}:=\emptyset$$;

$R_{I}^{-}:=\emptyset$; $R_{M}^{+}:=\emptyset$; $R_{M}^{-}:=\emptyset$; 

8.

CMI:=∅;

CMM:=∅;

Given a set of missing and wrong is-a relations and mappings for the ontology network, as an initial step we initialize knowledge bases for the ontology network (*KB*_*N*_, step 1), for each of the ontologies (*KB*_*k*_, step 2), and for each pair of ontologies with their alignments (*KB*_*ij*_, step 3). Then, we add all missing is-a relations (step 4) and mappings (step 5) to the relevant knowledge bases. As these are validated to be correct, this is extra knowledge that should be used in the repairing process. Adding the missing is-a relations and mappings essentially means that we have repaired these using the least informative repairing actions (see definition of more informative in the Additional file 1). In Subsection ‘Repairing missing is-a relations and mappings’ we try to improve this and find more informative repairing actions.

Further, we initialize global variables for the current sets of missing (MI) and wrong (WI) is-a relations, the current sets of missing (MM) and wrong (WM) mappings in step 6, the repairing actions ($R_{I}^{+}$ and $R_{I}^{-}$ for is-a relations, and $R_{M}^{+}$ and $R_{M}^{-}$ for mappings) in step 7, and the current sets of CMIs (CMI) and CMMs (CMM) in step.

#### Repairing wrong is-a relations and mappings

**Figure 3:**
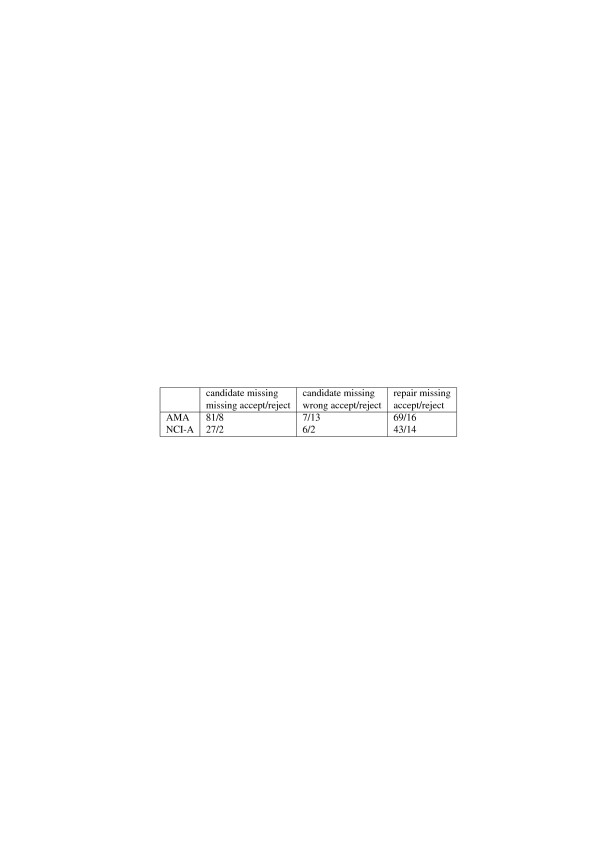
**An example of repairing wrong is-a relations. **Repairing wrong is-a relations: the case of *(brain grey matter, white matter)*, *(cerebellum white matter, brain grey matter) *and *(cerebral white matter, brain grey matter)*.

1. Compute

$$\text{AllJust}(w,r,O_{e})$$

where $O_{e}=(C_{e},I_{e})$ such that $C_{e}=\cup_{k=1}^{n}C_{k}$ and

$$\begin{array}{l} I_{e}=((\cup_{k=1}^{n}I_{k})\cup (\cup_{i,j=1;i<j}^{n}M_{\text{ij}})\cup {MI}_{N}\cup \\ {MM}_{N}\cup R_{I}^{+}\cup R_{M}^{+})\setminus (R_{I}^{-}\cup R_{M}^{-}) \end{array}$$

;

2. For every $I^{\prime }\in \text{AllJust}(w,r,O_{e})$:

 choose one element from $I^{\prime }\setminus ({MI}_{N}\cup {MM}_{N}\cup R_{I}^{+}\cup R_{M}^{+})$ to remove;

The algorithm for generating repairing actions for a wrong is-a relation or mapping is run for all wrong is-a relations (elements in WI) and mappings (elements in WM). It computes all justifications (AllJust) for the wrong is-a relation or mapping (w,r) in the current ontology network ($O_{e}$). The current network is the original network where the repairs up to now have been taken into account (i.e., all missing is-a relations are repaired by adding them while some are repaired using more informative repairing actions in $R_{I}^{+}$, missing mappings have been repaired by adding them or by repairing actions in $R_{M}^{+}$, and some wrong is-a relations and mappings are already repaired by removing is-a relations and mappings in $R_{I}^{-}$ and $R_{M}^{-}$, respectively). A justification for a wrong is-a relation or mapping can be seen as an explanation for why this is-a relation or mapping is derivable from the network (definition in Additional file 1). For instance, for the wrong is-a relation *(brain grey matter, white matter) *in AMA (experiment in Section ‘Experiment 1 - OAEI Anatomy’ and Figure 3), there is one justification: {*(brain grey matter (AMA), Brain_ White_Matter (NCI-A))*, *(Brain_ White_Matter (NCI-A), White_Matter (NCI-A))*, *(White_Matter (NCI-A), white matter (AMA))*}. In general, however, there may be several justifications for a wrong is-a relation or mapping. The algorithm to compute justifications initializes a knowledge base with the original ontology and the repairing actions up to now. To compute the justifications all derivation paths (see Additional file 1) are computed and the minimal ones are retained. The wrong is-a relation or mapping can then be repaired by removing at least one element in every justification. However, missing is-a relations, missing mappings, and added repairing actions (is-a relations in ontologies and mappings) cannot be removed. Using this algorithm structural repairs are generated that include only contributing repairing actions (pref1 in Subsection ‘Debugging workflow’).

When the repairing is executed, a number of updates need to be done. First, the wrong is-a relation (or mapping) is removed from WI (or WM). The chosen repairing actions that are is-a relations in an ontology are added to $R_{I}^{-}$ and repairing actions that are mappings are added to $R_{M}^{-}$. Some other wrong is-a relations or mappings may also have been repaired by repairing the current wrong is-a relation or mapping (update WI and WM). Also, some repaired missing is-a relations and mappings may also become missing again (update MI and MM). Further, new CMIs and CMMs may appear (update CMI and CMM - and after validation update CMI, MI, WI, CMM, MM and WM). In other cases the possible repairing actions for wrong and missing is-a relations and mappings may change (update justifications and sets of possible repairing actions for missing is-a relations and mappings). We also need to update the knowledge bases.

#### Repairing missing is-a relations and mappings

Repair missing is-a relation (a,b) with a $\in O_{k}$ and b $\in O_{k}$: Choose an element from GenerateRepairingActions(a, b, *KB*_*k*_);

Repair missing mapping (a,b) with a $\in O_{i}$ and b $\in O_{j}$:Choose an element from GenerateRepairingActions(a, b, *KB*_*ij*_);

GenerateRepairingActions(a, b, KB): 

1. *Source*(*a*,*b*) := super-concepts(*a*) - super-concepts(*b*)in KB;

2. *Target*(*a*,*b*) := sub-concepts(*b*) - sub-concepts(*a*) in KB;

3. *Repair*(*a*,*b*) := *Source*(*a*,*b*)×*Target*(*a*,*b*);

4. For each (*s*,*t*)∈*Source*(*a*,*b*)×*Target*(*a*,*b*):

 if $(s,t)\in WI\cup WM\cup R_{I}^{-}\cup R_{M}^{-}$ then remove (*s*,*t*) from *Repair*(*a*,*b*);

 else if $\exists (u,v)\in WI\cup WM\cup R_{I}^{-}\cup R_{M}^{-}:(s,t)$ is more informative than (*u*,*v*) in KB 

 and *u*→*s *and *t*→*v *are derivable from validated to be correct only is-a relations 

 and/or mappings

 then remove (*s*,*t*) from *Repair*(*a*,*b*); 

5. return *Repair*(*a*,*b*);

The algorithm for the computation of repairing actions for a missing is-a relation or mapping, an extension of the algorithm in [10], takes into consideration that all missing is-a relations and missing mappings will be repaired (least informative repairing action), but it does not take into account the consequences of the actual (possibly more informative) repairing actions that will be performed for other missing is-a relations and other missing mappings. The main component of the algorithm (GenerateRepairingActions) takes a missing is-a relation or mapping as input together with a knowledge base. For a missing is-a relation this is the knowledge base corresponding to the host ontology of the missing is-a relation; for a missing mapping this is the knowledge base corresponding to the host ontologies of the mapped concepts in the missing mapping and their alignment. In this component for a missing is-a relation or mapping we compute the more general concepts of the first concept (Source) and the more specific concepts of the second concept (Target) in the knowledge base. To avoid introducing non-validated equivalence relations where in the original ontologies and alignments there are only is-a relations, we remove the super-concepts of the second concept from Source, and the sub-concepts of the first concept from Target. Adding an element from Source × Target to the knowledge base makes the missing is-a relation or mapping derivable. However, some elements in Source × Target may conflict with already known wrong is-a relations or mappings. Therefore, in Repair, we take the wrong is-a relations and mappings and the former repairing actions for wrong is-a relations and mappings into account. The missing is-a relation or mapping can then be repaired using an element in Repair. We note that for missing is-a relations, the elements in Repair are is-a relations in the host ontology for the missing is-a relation. For missing mappings, the elements in Repair can be mappings as well as is-a relations in each of the host ontologies of the mapped concepts of the missing mapping. Using this algorithm structural repairs are generated that include only contributing repairing actions, and repairing actions of the form (*a*,*t*) or (*s*,*b*) for missing is-a relation or mapping (*a*,*b*) do not introduce non-validated equivalence relations (see pref1 and pref3 in Subsection ‘Debugging workflow’). Further, the solutions follow the single relations heuristic (pref4).

**Figure 4:**
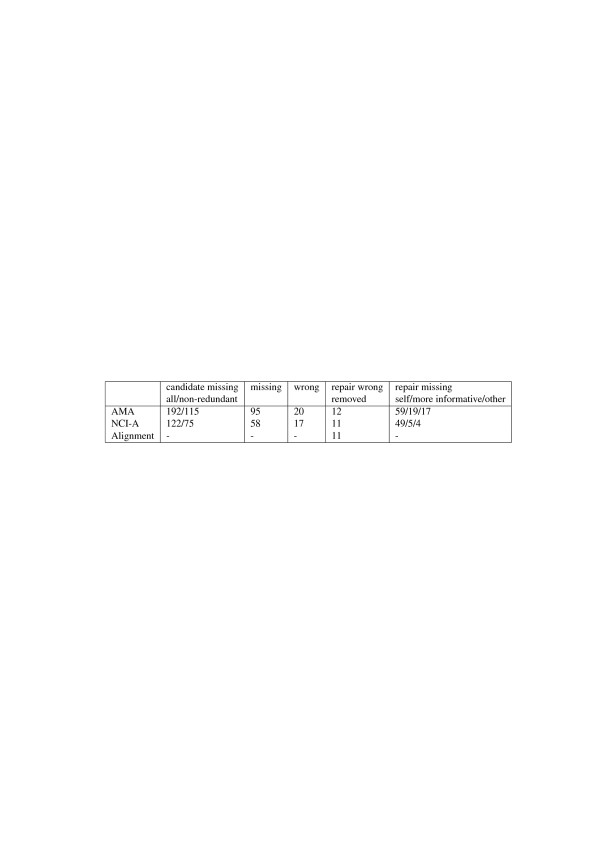
**An example of repairing missing is-a relations. **Repairing missing is-a relations: the case of *(lower respiratory tract cartilage, cartilage)*.

As an example, for the missing is-a relation *(lower respiratory track cartilage, cartilage) *in AMA (experiment in Section ‘Experiment 1 - OAEI Anatomy’ and Figure 4) a Source set of 2 elements and a Target set of 21 elements are generated and this results in 42 possible repairing actions. Each of the repairing actions when added to AMA, would make the missing is-a relation derivable from AMA. In this example a domain expert would select the more informative repairing action *(respiratory system cartilage, cartilage)*.

When the selected repairing action is in *Repair*(*a*,*b*), the repairing action is executed, and a number of updates need to be done. First, the missing is-a relation (or mapping) is removed from MI (or MM) and the chosen repairing action is added to $R_{I}^{+}$ or $R_{M}^{+}$ depending on whether it is an is-a relation within an ontology or a mapping. Further, new CMIs and CMMs may appear. Some other missing is-a relations or mappings may also have been repaired by repairing the current missing is-a relation or mapping. Some repaired wrong is-a relations and mappings may also become derivable again. In other cases the possible repairing actions for wrong and missing is-a relations and mappings may change. We also need to update the knowledge bases.

### Implemented system

#### Detecting and validating candidate missing is-a relations

**Figure 5:**
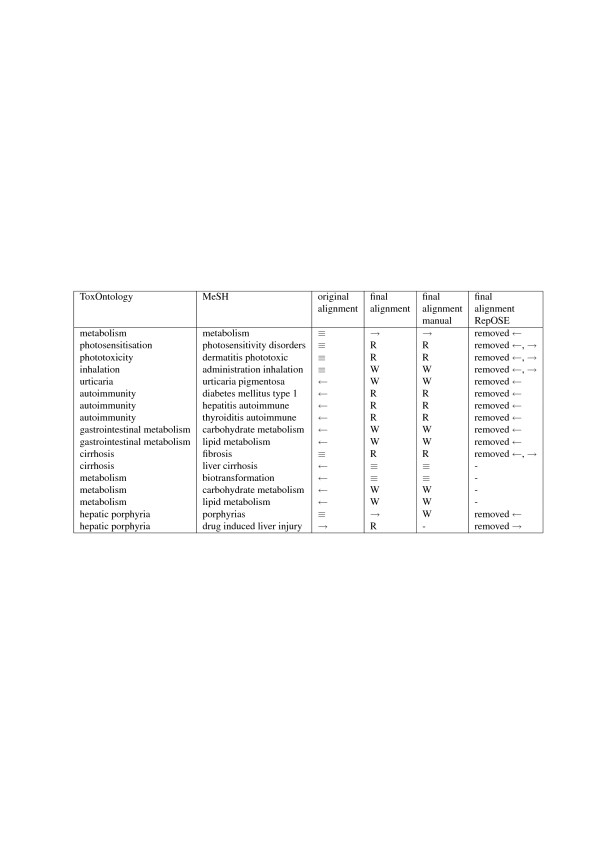
**An example of generating and validating candidate missing is-a relations.** Generating and validating candidate missing is-a relations: the case of *bone* and *joint*.

In RepOSE, the user loads the ontologies and alignments. Then the user can use the tab ‘Step1: Generate and Validate Candidate Missing is-a Relations’ (Figure 5) and choose an ontology for which the CMIs are computed. The user can validate all or some of the CMIs as well as switch to another ontology or another tab. Showing all CMIs at once would lead to information overload and difficult visualization. Showing them one at the time has the disadvantage that we do not have information about the interaction with other is-a relations. Therefore, as a trade-off we show the CMIs in groups where for each member of the group at least one of the concepts subsumes or is subsumed by a concept of another member in the group. Additionally, the CMIs can be chosen from a drop-down list. Initially, CMIs are shown using arrows labeled by ‘?’ (as in Figure 5 for *(acetabulum, joint)*) which the user can toggle to ‘W’ for wrong relations and ‘M’ for missing relations. For each CMI the justification in the ontology network is shown as an extra aid for the user. For instance, in Figure 5*(palatine bone, bone)* is selected and its justifications shown in the justifications panel. Concepts in different ontologies are presented with different background color. Further, we implemented a recommendation algorithm for validation. As is-a and part-of are often confused, the user can ask for a recommendation based on existing part-of relations in the ontology or in external domain knowledge (WordNet). If a part-of relation exists between the concepts of a CMI, it is likely a wrong is-a relation (the ‘?’ label is replaced by a ‘W?’ label as in Figure 5 for *(upper jaw, jaw)*). Similarly, the existence of is-a relations in external domain knowledge (WordNet and UMLS^g^) may indicate that a CMI is indeed a missing is-a relation (the ‘?’ label is replaced by a ‘M?’ label as in Figure 5 for *(elbow joint, joint)*). When a user decides to finalize the validation of a group of CMIs, RepOSE checks for contradictions in the current validation as well as with previous decisions and if contradictions are found, the current validation will not be allowed and a message window is shown to the user.

A similar tab ‘Step 2: Generate and Validate Candidate Missing Mappings’ can be used to choose a pair of ontologies and their alignment for which the CMMs are generated and, to validate them. The recommendation algorithm for mappings uses WordNet and UMLS.

#### Repairing wrong is-a relations and mappings

Figure 3 shows the RepOSE tab ‘Step 3: Repair Wrong is-a Relations’ for repairing wrong is-a relations. Clicking on the Generate Repairing Actions button, results in the computation of repairing actions for each wrong is-a relation of the ontology under repair. The wrong is-a relations are then ranked in ascending order according to the number of possible repairing actions and shown in a drop-down list. Then, the user can select a wrong is-a relation and repair it using an interactive display. The user can choose to repair all wrong is-a relations in groups or one by one. The display shows a directed graph representing the justifications. The nodes represent concepts. As mentioned before, concepts in different ontologies are presented with different background color. The concepts in the is-a relation under repair are shown in red. The edges represent is-a relations in the justifications. These is-a relations may be existing asserted is-a relations (shown in grey), mappings (shown in brown), unrepaired missing is-a relations (shown in blue) and the added repairing actions for the repaired missing is-a relations (shown in black).

In Figure 3 the user has chosen to repair several wrong is-a relations at the same time, i.e., *(brain grey matter, white matter)*, *(cerebellum white matter, brain grey matter)*, and *(cerebral white matter, brain grey matter)*. In this example we can repair these wrong is-a relations by removing the mappings between *brain grey matter* and *Brain_ White_Matter*. We note that, when removing these mappings, all these wrong is-relations will be repaired at the same time.

For the wrong is-a relations under repair, the user can choose, by clicking, multiple existing asserted is-a relations and mappings on the display as repairing actions and click the Repair button. RepOSE ensures that only existing asserted is-a relations and mappings are selectable, and when the user finalizes the repair decision, RepOSE ensures that the wrong is-a relations under repair and every selected is-a relation and mapping will not be derivable from the ontology network after the repairing. Further, all consequences of the repair are computed (such as changes in the repairing actions of other is-a relations and mappings and changes in the lists of wrong and missing is-a relations and mappings).

During the repairing, the user can choose to use the recommendation feature by enabling the Show Recommendation check box. The recommendation algorithm will then compute hitting sets for all the justifications of the wrong is-a relations under repair. Each hitting set contains a minimal set of is-a relations and mappings to remove so as to repair a wrong is-a relation (formal definition and algorithm in [28]). The recommendation algorithm then assigns a priority to each possible repairing action based on how often it occurs in the hitting sets and its importance in already repaired is-a relations and mappings. In the example in Figure 3 the highest priority (indicated by pink labels marked ‘Pn’, where n reflects the priority ranking) is given to the mapping *(Brain_ White_Matter, brain grey matter)*, as this is the only way to repair more than one wrong is-a relation at the same time. (Both *(cerebellum white matter, brain grey matter) *and *(cerebral white matter, brain grey matter) *would be repaired). Upon the selection of a repairing action, the recommendations are recalculated and the labels are updated. As long as there are labels, more repairing actions need to be chosen.

A similar tab (‘Step 4: Repair Wrong Mappings’) is used for repairing wrong mappings.

#### Repairing missing is-a relations and mappings

Figure 4 shows the RepOSE tab ‘Step 5: Repair Missing is-a Relations’ for repairing missing is-a relations. Clicking on the Generate Repairing Actions button, results in the computation of repairing actions for the missing is-a relations of the ontology under repair. For easy visualization, these are shown to the user as Source and Target sets (instead of Repair). Once the Source and Target sets are computed, the missing is-a relations are ranked with respect to the number of possible repairing actions. The first missing is-a relation in the list has the fewest possible repairing actions, and may therefore be a good starting point. When the user chooses a missing is-a relation, its Source and Target sets are displayed on the left and right, respectively, within the Repairing Actions panel (Figure 4). Both have zoom control and can be opened in a separate window. Similarly to the displays for wrong is-a relations, concepts in the missing is-a relations are highlighted in red, existing asserted is-a relations are shown in grey, unrepaired missing is-a relations in blue and added repairing actions for the missing is-a relations in black. For instance, Figure 4 shows the Source and Target sets for the missing is-a relation *(lower respiratory tract cartilage, cartilage)*, which contain 2 and 21 concepts, respectively. The Target panel shows also the unrepaired missing is-a relation *(nasal septum, nasal cartilage)*. The Justifications of current relation panel is a read-only panel that displays the justifications of the current missing is-a relation as an extra aid.

For the selected missing is-a relation, the user can also ask for recommended repairing actions by clicking the Recommend button. The recommendation algorithm (as defined in [10]) computes for missing is-a relation (*a*,*b*) the most informative repairing actions from *Source*(*a*,*b*) × *Target*(*a*,*b*) that are supported by domain knowledge (WordNet). In general, the system presents a list of recommendations. By selecting an element in the list, the concepts in the recommended repairing action are identified by round boxes in the panels. For instance, for the case in Figure 4, the recommendation algorithm proposes to add *(respiratory system cartilage, cartilage)*. Using the recommendation algorithm we recommend structural repairs that try to use as informative repairing actions as possible (pref2 in Subsection ‘Debugging workflow’). The user can repair the missing is-a relation by selecting a concept in the Source panel and a concept in the Target panel and clicking on the Repair button. When the selected repairing action is not in *Repair*(*a*,*b*), the repairing will not be allowed and a message window is shown to the user. Further, all consequences of a chosen repair are computed (such as changes in the repairing actions of other is-a relations and mappings and changes in the lists of wrong and missing is-a relations and mappings).

The tab ‘Step 6: Repair Missing Mappings’ is used for repairing missing mappings. The main difference with the tab for repairing missing is-a relations is that we deal with two ontologies and their alignment and that the repairing actions can be is-a relations within an ontology as well as mappings.

## Experiments

In this Section we discuss two debugging sessions. The first is a debugging session for two well-known ontologies in the anatomy domain. The second is work that we performed for the Swedish National Food Agency. In both cases the debugging was performed by domain experts. Further, we also discuss related work.

### Experiment 1 - OAEI Anatomy

In the first experiment a domain expert ran a complete debugging session on a network consisting of the two ontologies and the reference alignment from the Anatomy track in OAEI 2010. These ontologies as well as the reference alignment were developed by domain experts. For the 2010 version of OAEI, AMA contains 2,744 concepts and 1,807 asserted is-a relations, while NCI-A contains 3,304 concepts and 3,761 asserted is-a relations. The reference alignment contains 986 equivalence and 1 subsumption mapping between AMA and NCI-A. This information is summarized in Table 1.

**Table 1:** Experiment 1 - ontologies and alignment

	**Concepts**	**Asserted is-a relations**	**Asserted equivalence mappings**	**Asserted is-a mappings**
AMA	2744	1807	-	-
NCI-A	3304	3761	-	-
Alignment	-	-	986	1

The experiment was performed on an Intel Core i7-950 Processor 3.07GHz with 6 GB DDR2 memory under Windows 7 Ultimate operating system and Java 1.7 compiler. The domain expert completed debugging this network within 2 days. As the system has a good responsiveness, much of this time was spent on making decisions for validation and repairing (essentially looking up and analyzing information to make decisions) and interactions with RepOSE.

Table 2 summarizes the results of the detection and repairing of defects in the is-a structures of the ontologies and the mappings. The system detected 200 CMIs in AMA of which 123 were non-redundant. Of these non-redundant CMIs 102 were validated to be missing is-a relations and 21 were validated to be wrong is-a relations. For NCI-A 127 CMIs, of which 80 non-redundant, were detected. Of these non-redundant CMIs 61 were validated to be missing is-a relations and 19 were validated to be wrong is-a relations. To repair these defects 85 is-a relations were added to AMA and 57 to NCI-A, 13 is-a relations were removed from AMA and 12 from NCI-A, and 12 mappings were removed from the reference alignment. In 22 cases in AMA and 8 cases in NCI-A a missing is-a relation was repaired using a more informative repairing action, thereby adding new knowledge to the network.

**Table 2:** Experiment 1 results - final result

	**Candidate missing**	**Missing**	**Wrong**	**Added**	**Repair missing**	**Removed**	**Removed**
	**all/non-redundant**			**is-a relations**	**more informative**	**is-a relations**	**mappings**
AMA	200/123	102	21	85	22	13	-
NCI-A	127/80	61	19	57	8	12	-
Alignment	-	-	-	-	-	-	12

The ranking and recommendations seemed useful. Table 3 summarizes the recommendation results. Regarding CMIs, 81 and 27 recommendations that the relation should be validated as a missing is-a relation, were accepted for AMA, respectively NCI-A, while 8 and 2 were rejected. When the system recommended that a CMI should be validated as a wrong is-a relation, the recommendation was accepted in 7 out of 20 cases for AMA and 6 out of 8 cases for NCI-A. The recommendations regarding repairing missing is-a relations were accepted in 69 out of 85 cases for AMA and 43 out of 57 cases for NCI-A. We note that the system may not always give a recommendation. This is the case, for instance, when there is no information about the is-a relation under consideration in the external sources.

**Table 3:** Experiment 1 results - recommendations

	**Candidate missing**	**Candidate missing**	**Repair missing**
	**missing accept/**	**wrong accept/**	**accept/reject**
	**reject**	**reject**	
AMA	81/8	7/13	69/16
NCI-A	27/2	6/2	43/14

In the remainder of this Subsection we discuss the session and the results in more details.

#### Detecting and validating candidate missing is-a relations for the first time

After loading AMA, NCI-A and the reference alignment, it took less than 30 seconds for each of the ontologies to detect all its CMIs. As a result, RepOSE found 192 CMIs in AMA and 122 in NCI-A. Among these CMIs, 115 in AMA and 75 in NCI-A are displayed in 24 groups and 18 groups, respectively, for validation, while the remaining 77 in AMA and 47 in NCI-A are redundant and thus ignored. With the help of the recommendations, the domain expert identified 20 wrong is-a relations and 95 missing is-a relations in AMA. For NCI-A the domain expert identified 17 wrong and 58 missing is-a relations. These results are summarized in Table 4. As for the recommendation, the use of asserted part-of relations in ontologies together with WordNet recommended 20 possible wrong is-a relations in AMA and 8 in NCI-A, of which 7 in AMA and 6 in NCI-A were accepted as decisions. WordNet and UMLS recommended 84 possible missing is-a relations in AMA and 29 in NCI-A, of which 77 in AMA and 27 in NCI-A were accepted as decisions.

**Table 4:** Experiment 1 results - first iteration

	**Candidate missing**	**Missing**	**Wrong**	**Repair wrong**	**Repair missing**
	**all/non-redundant**			**removed**	**self/more informative/other**
AMA	192/115	95	20	12	59/19/17
NCI-A	122/75	58	17	11	49/5/4
Alignment	-	-	-	11	-

#### Repairing wrong is-a relations for the first time

After the validation phase, the domain expert continued with the repairing of wrong is-a relations. In this experiment, for the 20 wrong is-a relations in AMA and 17 in NCI-A, each wrong is-a relation has only one justification, consisting of two or more mappings and one or more asserted is-a relations in the other ontology. Therefore, the repairing is done by removing the involved asserted is-a relations and/or mappings (Table 4). For example, for the wrong is-a relation *(Ascending_Colon, Colon)* in NCI-A (which is actually a part-of relation), its justification contains two equivalence mappings (between *Ascending_Colon *and *ascending colon*, and between *Colon *and *colon*) and an asserted is-a relation *(ascending colon, colon) *in AMA. The repairing was done by removing *(ascending colon, colon) *from AMA. As shown before, the wrong is-a relation *(brain grey matter, white matter) *in AMA (Figure 3) was repaired by removing the mappings between *Brain_White_Matter *and *brain grey matter*.

We note that 11 mappings were removed, 8 of them as a result of wrong is-a relations in AMA and 3 as a result of the debugging of NCI-A. Further, several wrong is-a relations were repaired by repairing other wrong is-a relations.

#### Repairing missing is-a relations in AMA and NCI-A for the first time

As the next step, the domain expert proceeded with the repairing of missing is-a relations in AMA. At this point there were 95 missing is-a relations to repair, and it took less than 10 seconds to generate the repairing actions for them. Almost all Source and Target sets were small enough to allow a good visualization. For 59 missing is-a relations, the domain expert used the missing is-a relation itself as the repairing action (i.e., the least informative repairing actions). For 19 missing is-a relations, the domain expert used more informative repairing actions, which also repaired 17 other missing is-a relations. These results are summarized in the last column of Table 4. The recommendation algorithm was used in 78 cases. In 63 of them the selected repairing action was among the recommended repairing actions and in 9 of them the recommendation algorithm suggested more informative repairing actions.

The domain expert then continued with the repairing of missing is-a relations in NCI-A. For the 58 missing is-a relations to repair, 49 missing is-a relations were repaired using themselves as the repairing actions, 5 were repaired using more informative repairing actions, and 4 were repaired by the repairing of others (Table 4). For example, for the repairing of missing is-a relation *(Epiglottic_Cartilage, Laryngeal_Connective_Tissue) *in NCI-A, the domain expert used more information repairing action *(Laryngeal_Cartilage, Laryngeal_Connective_Tissue)*, where *Laryngeal_Cartilage *is a super-concept of *Epiglottic_Cartilage *in NCI-A. This repairing also repaired 3 other missing is-a relations, i.e., *(Cricoid_Cartilage, Laryngeal_Connective_Tissue)*, *(Arytenoid_Cartilage, Laryngeal_Connective_Tissue) *and *(Thyroid_Cartilage, Laryngeal_Connective_Tissue)*, where *Cricoid_Cartilage*, *Arytenoid_Cartilage *and *Thyroid_Cartilage *are sub-concepts of *Laryngeal_Cartilage *in NCI-A. The recommendation algorithm was used in 54 cases. In 42 of them the selected repairing action was among the recommended repairing actions and in 3 of them the recommendation algorithm suggested more informative repairing actions.

We observe that at this point for 19 missing is-a relations in AMA and 5 in NCI-A, the domain expert has used repairing actions that are more informative than the missing is-a relation itself. This means that for each of these the domain expert has added knowledge that was not intrinsic to (i.e., derivable from) the network. Thus the knowledge represented by the ontologies and the network has increased.

#### The subsequent debugging process

The repairing of the wrong and the missing is-a relations in both ontologies resulted in 6 non-redundant new CMIs in AMA and 4 in NCI-A. In each ontology 1 of those was validated as wrong and the others as missing. 2 of the 5 missing is-a relations in AMA were repaired by themselves and 3 using more informative repairing actions. The wrong is-a relation was repaired by removing an is-a relation in NCI-A. The 3 missing is-a relations in NCI-A were repaired by using more informative repairing actions. The wrong is-a relation was repaired by removing a mapping from the reference alignment. The repairing of these newly found relations led to two more CMIs in AMA, which were validated as correct and repaired by themselves, and one CMI in NCI-A, which was validated as wrong and repaired by removing an is-a relation in AMA. At this point there were no more CMIs to validate, and no more wrong or missing is-a relations to repair.

### Experiment 2 - ToxOntology, MeSH and their alignment

In this Subsection we describe a debugging session that was performed for and with the Swedish National Food Agency [29]. As part of an initiative to facilitate adequate identification and display of substance-associated health effects a toxicological ontology - ToxOntology - was created. ToxOntology is an OWL2 ontology, encompassing 263 concepts and 266 asserted is-a relations. The task was to help the Swedish National Food Agency to create an alignment with MeSH and debug the ontologies.

As MeSH contains many descriptors not related to the domain of toxicology, we used parts from the Diseases [C], Analytical, Diagnostic and Therapeutic Techniques and Equipment [E] and Phenomena and Processes [G] branches of MeSH. Further, the hierarchical relation in MeSH does not necessarily represent the is-a relation. For this experiment we therefore created an ontology (which we will call MeSH in this experiment) that contains 9,878 concepts which are related to descriptors in the [C], [E] and [G] branches and 15,786 asserted is-a relations which relate to hierarchical relations. We note that the asserted is-a relations therefore may not always be correct with respect to the domain.

#### Aligning ToxOntology and MeSH

Mapping suggestions were created using our ontology alignment system SAMBO [30]. During the validation phase a domain expert classified the mapping suggestions into: equivalence mapping, is-a mapping (ToxOntology term is-a MeSH term and MeSH term is-a ToxOntology term), related terms mapping and wrong mapping. The resulting validated alignment consists of 41 equivalence mappings, 43 is-a mappings between a ToxOntology term and a MeSH term, 49 is-a mappings between a MeSH term and a ToxOntology term and 243 related terms mappings. Further, there is information about 1136 wrong mappings.

#### Debugging using validated alignment

It was not considered feasible to identify defects manually. Therefore, we used the detection mechanisms of RepOSE. RepOSE computed CMIs, which were then validated by domain experts. As there initially were only 29 CMIs, we decided to repair the ontologies and their alignment independently in two ways. First, the CMIs and their justifications were given to the domain experts who manually repaired the ontologies and their alignment. Second, the repairing mechanisms of RepOSE were used.

A summary of the changes in the alignment and in ToxOntology due to the debugging sessions are summarized in Table 5 columns ‘original alignment’ and ‘final alignment’, and Table 6 column ‘final’, respectively. There are also 5 missing is-a relations for MeSH. In the remainder of this Subsection we describe the detection and repairing in more details and compare the manual repairing with the repairing using RepOSE.

**Table 5:** Experiment 2 - Changes in the alignment (equivalence mapping (≡), ToxOntology term is-a MeSH term (→), MeSH term is-a ToxOntology term (←), related terms (R), wrong mapping (W))

**ToxOntology**	**MeSH**	**Original**	**Final**	**Final**	**Final**
		**alignment**	**alignment**	**alignment**	**alignment**
				**manual**	**RepOSE**
Metabolism	Metabolism	≡	→	→	removed ←
Photosensitisation	Photosensitivity disorders	≡	R	R	removed ←, →
Phototoxicity	Dermatitis phototoxic	≡	R	R	removed ←, →
Inhalation	Administration inhalation	≡	W	W	removed ←, →
Urticaria	Urticaria pigmentosa	←	W	W	removed ←
Autoimmunity	Diabetes mellitus type 1	←	R	R	removed ←
Autoimmunity	Hepatitis autoimmune	←	R	R	removed ←
Autoimmunity	Thyroiditis autoimmune	←	R	R	removed ←
Gastrointestinal metabolism	Carbohydrate metabolism	←	W	W	removed ←
Gastrointestinal metabolism	Lipid metabolism	←	W	W	removed ←
Cirrhosis	Fibrosis	≡	R	R	removed ←, →
Cirrhosis	Liver cirrhosis	←	≡	≡	-
Metabolism	Biotransformation	←	≡	≡	-
Metabolism	Carbohydrate metabolism	←	W	W	-
Metabolism	Lipid metabolism	←	W	W	-
Hepatic porphyria	Porphyrias	≡	→	W	removed ←
Hepatic porphyria	Drug induced liver injury	→	R	-	removed →

**Table 6:** Experiment 2 - Changes in the structure of ToxOntology

**Added is-a relations**	**Final**	**Manual**	**RepOSE**
Absorption → physicochemical parameter	Yes	Yes	Yes
Hydrolysis → metabolism	Yes	Yes	Yes
Toxic epidermal necrolysis → hypersensitivity	Yes	Yes	Yes
Urticaria → hypersensitivity	Yes	Yes	Yes
Asthma → hypersensitivity	Yes	Yes	Yes
Asthma → respiratory toxicity	Yes	Yes	No
Allergic contact dermatitis → hypersensitivity	Yes	Yes	Yes
Subcutaneous absorption → dermal absorption	Yes	Yes	Yes
Oxidation → metabolism	Yes	Yes	Yes
Oxidation → physicochemical parameter	Yes	Yes	Yes

#### Detection using RepOSE

As input to RepOSE we used ToxOntology and the part of MeSH described earlier. Further, we used the validated part of the alignment discussed in Section ‘Aligning ToxOntology and MeSH’, that contains the 41 equivalence mappings, the 43 is-a mappings between a ToxOntology term and a MeSH term and the 49 is-a mappings between a MeSH term and a ToxOntology term.^h ^RepOSE generated 12 non-redundant CMIs for ToxOntology (34 in total) of which 9 were validated by the domain experts as missing and 3 as wrong. For MeSH, RepOSE generated 17 non-redundant CMIs (among which 2 relations represented one equivalence relation - 32 CMIs in total) of which 5 were validated as missing and the rest as wrong.

#### Manual repair

The domain experts focused on repairment of ToxOntology and the alignment. Regarding the 9 missing is-a relations in ToxOntology, these were all added to the ontology. Further, another is-a relation, *(asthma, respiratory toxicity*), was added, in addition to *(asthma, hypersensitivity)*, based on an analogy of this case with the already existing is-a relation *(urticaria, dermal toxicity) *and the added is-a relation *(urticaria, hypersensitivity)*. This is summarized in Table 6 column ‘manual’. The domain experts also removed two asserted is-a relations (*(asthma, immunotoxicity) *and *(subcutaneous absorption, absorption)*) for reasons of redundancy. These is-a relations are valid and they are derivable in ToxOntology.

The wrong is-a relations for MeSH and ToxOntology were all repaired by removing mappings in the alignment (Table 5 column ‘final alignment manual’). In 5 cases a mapping was changed from equivalence or is-a into related. In one of the cases (concerning *cirrhosis *in ToxicOntology and *fibrosis *and *liver cirrhosis *in MeSH) a further study led to the change of *cirrhosis *←*liver cirrhosis* into *cirrhosis *≡ *liver cirrhosis*.

The wrong is-a relations involving *metabolism *in ToxOntology, invoked a deeper study of the use of this term in ToxOntology and in MeSH. The domain experts concluded that the ToxOntology term *metabolism *is equivalent to the MeSH term *biotransformation *and a sub-concept of the MeSH term *metabolism*. This observation led to a repair of the mappings related to *metabolism*.

Further, some mappings were changed from an equivalence or is-a mapping to a wrong mapping. In these cases (e.g., between *urticaria *in ToxOntology and *urticaria pigmentosa *in MeSH) the terms were syntactically similar and were initially validated wrongly during the alignment phase.

#### Repairing using RepOSE

In this second way of repairing the domain expert used RepOSE. For the 9 missing is-a relations in ToxOntology and the 5 missing is-a relations in MeSH, possible repairing actions (using Source and Target sets) were generated. For most of these missing is-a relations the Source and Target sets were small, although for some there were too many elements in the set to provide for good visualization. For all these missing is-a relations the repairing constituted of adding the missing is-a relations themselves (Table 6 column ‘RepOSE’). In all but three cases this is what RepOSE recommended based on external knowledge from WordNet and UMLS. In 3 cases the system recommended to add other is-a relations, that were not considered correct by the domain experts (and thus wrong or based on a different view of the domain in the external domain knowledge).

For the 3 wrong is-a relations for ToxOntology and the 12 wrong is-a relations for MeSH, the justifications were shown to the domain experts. The justifications for a wrong is-a relation contained at least 2 mappings and 0 or 1 is-a relations in the other ontology. In each of these cases the justification contained at least one mapping that the domain expert validated to be wrong or related and the wrong is-a relations were repaired by removing these mappings (see Table 5 column ‘final alignment RepOSE’, except last row). In some cases repairing one wrong is-a relation also repaired others (e.g., removing mapping *hepatic porphyria *←*porphyrias*, repairs two wrong is-a relations in MeSH: *(porphyrias, porhyrias hepatic) *and *(porphyrias, drug induced liver injury)*).

After this repairing, we detected one new CMI in MeSH. This was validated as a wrong is-a relation and resulted in the removal of one more mapping (see Table 5 column ‘final alignment RepOSE’ last row).

#### Debugging using non-validated alignment

In the previous Subsection the validated alignment was used as input. As a domain expert validated the mappings, they could be considered of high quality, although we showed that defects in the mappings were detected. In this Subsection we discuss an experiment with a non-validated alignment; we used the 41 mapping suggestions generated by SAMBO with a similarity value higher than or equal to 0.8 and used them initially as equivalence mappings.^i^

Using RepOSE (in 2 iterations) 16 non-redundant CMIs (27 in total), were computed for ToxOntology of which 6 were also computed in the debugging session described earlier. For MeSH 6 non-redundant CMIs (10 in total) were computed of which 2 were also computed earlier. As expected, the newly computed CMIs were all validated as wrong is-a relations and their computation was a result of wrong mappings. During the repairing 5 of the 7 wrong mappings were removed, and 2 initial mappings were changed into is-a mappings. RepOSE can thus be helpful in the validation of non-validated alignments - a domain expert will be able to remove wrong mappings that lead to wrong is-a relations, but other wrong mappings may not be found.

## Discussion and related work

### Discussion

Generally, detecting defects in ontologies without the support of a dedicated system is cumbersome and unreliable. According to the domain experts, in the cases outlined in this paper RepOSE clearly provided a necessary support. Further, visualization of the justifications of possible defects was very helpful to have at hand as well as a graphical display of the possible defects within their contexts in the ontologies. Moreover, RepOSE stored information about all changes made and their consequences as well as the remaining defects needing amendment.

As the set of CMIs in the second experiment was relatively small, it was possible for domain experts to perform a manual repair. They could focus on the pieces of ToxOntology that were related to the missing and wrong is-a relations. This allowed us to compare results of manual repair with those of repairment using RepOSE. Regarding the changes in the alignment, for 11 term pairs the mapping was removed or changed in both approaches. For 2 term pairs the manual approach changed an is-a relation into an equivalence and for 2 other term pairs an is-a relation was changed into a wrong relation. These changes were not logically derivable and could not be found by RepOSE. For 3 of these term pairs the change came after the domain experts realized (using the justifications of the CMIs) that *metabolism *in MeSH has a different meaning than *metabolism *in ToxOntology. For 1 term pair (one but last row in Table 5) the equivalence mapping was changed into a wrong mapping by the domain experts, while using RepOSE it was changed into an is-a relation. In the final alignment the RepOSE result was used. Further, through a second round of detection, using RepOSE an additional wrong mapping was detected and repaired, which was not found in the manual repair. Regarding the addition of is-a relations to ToxOntology, the domain experts added one more is-a relation in the manual approach than in the approach using RepOSE. It could not be logically derived that *(asthma, respiratory toxicity) *was missing, but it was added by the domain experts in analogy to the repairing of another missing is-a relation.

In some cases, when using RepOSE, the justification for a missing is-a relation was removed after a wrong is-a relation was repaired by removing a mapping. For instance, after removing *metabolism (ToxicOntology) ← metabolism (MeSH) *in the second experiment, there was no more justification for the missing is-a relation *(hydrolysis, metabolism)*. However, one advantage of RepOSE is that once a relation is validated as missing, RepOSE requires that it be repaired and thus, this knowledge will be added, even without a justification.

Another advantage of RepOSE is that, for repairing a wrong is-a relation, it allows to remove multiple is-a relations and mappings in the justification, even though sometimes it is sufficient to remove one. This was used, for instance, in the repair of the wrong is-a relation *(phototoxicity, photosensitisation) *in ToxOntology where *photosensitisation ≡ photosensitivity disorders *and *phototoxicity ≡ dermatitis phototoxic *were removed. Further, the repairing of one defect can lead to other defects being repaired. For instance, the removal of these two mappings also repaired the wrong is-a relation *(photosensitivity disorders, dermatitis phototoxic) *in MeSH. In general, RepOSE facilitates the computation and understanding of the consequences of repairing actions.

The repairing of is-a relations in the two experiments was quite different. To repair wrong is-a relations, in the second experiment only mappings were removed. This indicates that the ontology developers modeled the is-a structure decently. In the first experiment, however, 25 is-a relations and 12 mappings were removed. To repair missing is-a relations, in the second experiment all missing is-a relations were repaired by adding the missing is-a relations themselves. In the first experiment, however, in 30 cases a missing is-a relation was repaired using a more informative repairing action, thereby adding new knowledge that was not derivable from the ontologies and their alignment.

As we need at least 3 ontologies and 2 alignments to find CMMs, there were no such in the discussed experiments. In another experiment [31] in the bibliography domain with 5 ontologies and 4 alignments, our approach found CMIs and CMMs and the repairing was done by adding and removing is-a relations and mappings. Further, we note that in this setting RepOSE can also be used to align ontologies. Indeed, in the bibliography experiment alignments were generated for each pair of ontologies for which no alignment existed previously.

Our approach for detection CMIs and CMMs is logic-based. The advantage of this approach is that it is guaranteed that all missing is-a relations and mappings derivable from the network will be found after a number of iterations. A disadvantage is the fact that we need is-a relations in the ontologies. In the second experiment we used the hierarchy in MeSH as if it represents is-a relations although, in general, it does not. Therefore, some identified wrong is-a relations in MeSH may actually not be is-a relations in MeSH.

Another constraint of RepOSE pertains to the fact that adding and removing is-a relations and mappings not appearing in the computations in RepOSE can be a demanding undertaking. Currently, these changes need to be conducted in the ontology files, but it would be useful to allow a user to do this via the system. For instance, in the second experiment it would have been useful to add *(asthma, respiratory toxicity) *via RepOSE. An integration of RepOSE functionality and functionality of ontology development systems would solve such issues.

### Related work

The approach in this paper is an extension of our previous work on debugging missing is-a relations in single taxonomies [10] where we assumed that the existing is-a structure is correct. This work was extended in [11] where we dealt with missing and wrong is-a relations in taxonomies in a network. The assumption in that work was that the existing mappings were correct. In this work we extended the approach to a unified approach that deals with missing and wrong is-a relations as well as with missing and wrong mappings and their interactions.

There is not much work on debugging modeling defects in networked ontologies. The work closest to our own regarding missing is-a relations is [9], where a similar method for detection is used and a validation of the results is also needed. However, repair consists only of adding the missing is-a relations. The work in [32] discusses the alignment of AMA and NCI-A and uses the notion of structural validation to remove mappings that cannot be structurally validated. Structural validation could be used to detect candidate missing is-a relations. In [33] we showed that the problem of repairing missing is-a relations can be formalized as an abduction problem.

In [8] the authors propose an approach for detecting modeling and semantic defects within an ontology based on patterns and antipatterns. The patterns and antipatterns are logic-based and mainly deal with logical constructs not available in taxonomies. Some suggestions for repairing are also given. In [34] a pattern-based approach is presented for the detection of wrong and missing mappings. The approach was tested on the OAEI Anatomy 2010 data and the results incorporated in the OAEI Anatomy 2011 data. We have compared the results of our approach with the results of [34] regarding wrong mappings. Of the 25 wrong mappings identified by [34], our approach can identify 21 wrong mappings using the full^j^ reference alignment. Our approach identified, furthermore, 8 additional wrong mappings.

There is more work that addresses semantic defects in ontologies. Most of it aims at identifying and removing logical contradictions from an ontology. Standard reasoners are used to identify the existence of a contradiction, and provide support for resolving and eliminating it [35]. In [7] minimal sets of axioms are identified which need to be removed to render an ontology coherent. An algorithm for finding solutions is proposed which uses a variant of the single relation heuristic. Similarly, in [5,6] strategies are described for repairing unsatisfiable concepts detected by reasoners, explanation of errors, ranking erroneous axioms, and generating repair plans. The generated solutions, however, are based on other heuristics than [7] and our work. In [36] the focus is on maintaining the consistency as the ontology evolves through a formalization of the semantics of change for ontologies. In [13-15] the setting is extended to repairing ontologies connected by mappings. In this case, semantic defects may be introduced by integrating ontologies. All approaches assume that ontologies are more reliable than the mappings and try to remove some of the mappings to restore consistency. In [13,15] the solutions are based on the computation of minimal unsatisfiability-preserving sets or minimal conflict sets. While [13] proposes solutions based on a heuristic using distance in WordNet, [15] allows the user to choose between all, some or one solution. In [14] the authors focus on the detection of certain kinds of defects and redundancy. The work in [37] further characterizes the problem as mapping revision. Using belief revision theory, the authors give an analysis for the logical properties of the revision algorithms. The approach in [12] deals with the inconsistencies introduced by the integration of ontologies, and unintended entailments validated by the user. We note that most of these approaches can deal with ontologies represented in more expressive languages than in our work. However, few approaches have implemented systems and the approaches are usually only tested on small ontologies.

A different setting is the area of modular ontologies where the ontologies are connected by directional mappings and where knowledge propagation only occurs in one direction. Regarding the detection of semantic defects, within a framework based on distributed description logics, it is possible to restrict the propagation of local inconsistency to the whole set of ontologies (e.g., [38]).

Related to the detection of missing relations, there is much work on finding relationships between terms in the ontology learning area [39]. In this setting, new ontology elements are derived from text using knowledge acquisition techniques. Regarding the discovery of subsumption relations, one paradigm is based on linguistics using lexico-syntactic patterns. The pioneering research conducted in this line is in [40], which defines a set of patterns indicating is-a relationships between words in the text. Another paradigm is based on machine learning and statistical methods. Compared with these approaches, our detection method uses the ontology network as the domain knowledge for the discovery of is-a relations. It is able to deal with multiple ontologies at the same time rather than a single ontology. However, these approaches are complementary to our detection method, in that, results from them, after validation, could be used as input to the repairing phase.

## Conclusions

To obtain satisfactory results in the semantically-enabled applications, high-quality ontologies and alignments are both necessary. A key step towards this is debugging the ontologies and their alignments. In this paper we have proposed an approach for debugging the is-a structure of ontologies and mappings between ontologies in a network of taxonomies. We defined important notions and developed algorithms for detection and repair of the missing and wrong is-a structure and mappings. We also implemented a system and showed the usefulness of the approach through two experiments.

We note that the repairing algorithms can also be used for single ontologies when initial sets of missing and wrong is-a relations are given. Further, the detection phase in the framework can easily be extended to approaches using external knowledge to detect missing and wrong is-a relations and mappings. A first interesting direction for future work is therefore to integrate such approaches with RepOSE. For instance, we intend to use our ontology alignment system SAMBO [30], that will provide candidate missing mappings.

Another connection between ontology debugging and ontology alignment is given in [21] where one of the ontology alignment approaches includes detecting missing is-a relations by using the structure of the ontologies and a set of correct mappings. The missing is-a relations were repaired by adding them to the ontologies before starting the actual alignment process. In the future we intend to study this interaction between ontology debugging and ontology alignment in a deeper way.

A further interesting direction is to deal with ontologies represented in more expressive representation languages. The techniques described in this paper may be partly used for these ontologies, but a number of conditions (such as the consequences of negation and disjointness) will need to be taken into account. We also want to study the interaction between repairing actions. For instance, it may be more important to repair the top level in the ontology first and in this case, the ranking approach should reflect this.

## Endnotes

^a^These mappings may be generated by an ontology alignment system or manually created by a domain expert. In general, each mapping may be correct or wrong according to the intended model of the domains. One of the aims of this paper is to detect and repair the wrong mappings.

^b^The first ontology is a part of AMA, the second ontology is a part of NCI-A, and the alignment is a part of the alignment between AMA and NCI-A as defined in OAEI 2010.

^c^We note that the repairing does not require that the missing and wrong is-a relations and mappings be determined using the technique for detection described above. They may have been generated using external knowledge (e.g., by an ontology alignment system for the mappings) and then validated by a domain expert or they may have been provided directly by a domain expert.

^d^We also note that using *(viscerocranium bone, bone) *as repairing action would also immediately repair the missing is-a relations *(maxilla, bone) *and *(lacrimal bone, bone)*.

^e^In the worst case scenario the number of mapped concept pairs is equal to the total number of concept pairs. In practice, the use of mapped concepts may significantly reduce the search space, e.g., when some ontologies are smaller than other ontologies in the network or when not all concepts participate in mappings. For instance, in the experiment in Section ‘Experiment 1 - OAEI Anatomy’ the search space is reduced by almost 90%.

^f^See Additional file 1 for our definition of knowledge base as well as the initialization.

^g^It is well-known that UMLS contains semantic and modeling defects (e.g., [41,42]). Therefore, we only use the external resources in the *recommendation *of the validation of CMIs (and in Section ‘Repairing missing is-a relations and mappings’ in the recommendation of repairing actions), but not in the *generation*. The validation (and in Section ‘Repairing missing is-a relations and mappings’ the choice of repairing actions) is always the domain expert’s responsibility and the recommendations should only be considered as an aid.

^h^The related terms mappings cannot be used in logical derivation related to the is-a structure of the ontologies and are therefore not included in the alignment used in RepOSE.

^i^From the validation we know that these actually contain 29 equivalence mappings, 2 is-a mappings between a ToxOntology term and a MeSH term, 2 is-a mappings between a MeSH term and a ToxOntology term, 1 related terms mapping and 7 wrong mappings.

^j^The reference alignment in experiment 1 is a partial alignment. The full reference alignment was not available at the time of the experiment.

## Competing interests

Both authors declare that they have no competing interests.

## Authors’ contributions

PL defined most of the theory. VI did the implementation work. The other tasks were performed by both authors. Both authors read and approved the final manuscript.

## Supplementary Material

Additional file 1 Supplementary material. Click here for file
