# Supplementary material for: A unified approach for debugging is-a structure and mappings in networked taxonomies
Source: J Biomed Semantics. 2013 Mar 31;4:10. doi: 10.1186/2041-1480-4-10 (PMC3851282; doi:10.1186/2041-1480-4-10)
Supplement: Additional file 1 — Supplementary material. [file 2041-1480-4-10-S1.pdf]

Supplementary material

Patrick Lambrix*[1] and Valentina Ivanova[1]

[1]Department of Computer and Information Science / Swedish e-Science Research Centre, Linköping University, 581 83 Linköping, Sweden

Email: Patrick Lambrix*- Patrick.Lambrix@liu.se; Valentina Ivanova - Valentina.Ivanova@liu.se;

*Corresponding author

In this supplementary material we formalize the notions and provide proofs.

2. Methods

2.1 Preliminaries

The ontologies that we study are taxonomies, which are defined using named concepts and subsumption axioms (is-a relations between concepts). For this paper we use the following definition.

Definition 1 *An* **ontology** $\mathcal{O}$ *is represented by a tuple* $(\mathcal{C}, \mathcal{I})$ *where* $\mathcal{C}$ *is its set of named concepts and* $\mathcal{I} \subseteq \mathcal{C} \times \mathcal{C}$ *is a set of asserted is-a relations, representing the is-a structure of the ontology.*

The ontologies are connected into a network through alignments which are sets of mappings between concepts from two different ontologies. We currently consider mappings of the type *equivalent* ($\equiv$), *subsumed-by* ($\rightarrow$) and *subsumes* ($\leftarrow$).

Definition 2 *An* **alignment** *between ontologies* $\mathcal{O}_i$ *and* $\mathcal{O}_j$ *is represented by a set* $\mathcal{M}_{ij}$ *of pairs representing the* **mappings**, *such that for concepts* $c_i \in \mathcal{O}_i$ *and* $c_j \in \mathcal{O}_j$: $c_i \rightarrow c_j$ *is represented by* (c_i, c_j); $c_i \leftarrow c_j$ *is represented by* (c_j, c_i); *and* $c_i \equiv c_j$ *is represented by both* (c_i, c_j) *and* (c_j, c_i).[1]

The concepts that participate in mappings we call **mapped concepts**. Concepts can participate in multiple mappings.

Definition 3 *An* **ontology network** $\mathcal{N}$ *is a tuple* $(\mathbb{O}, \mathbb{M})$ *with* $\mathbb{O} = \{\mathcal{O}_k\}_{k=1}^n$ *the set of the ontologies in the network and* $\mathbb{M} = \{\mathcal{M}_{ij}\}_{i,j=1;i<j}^n$ *the set of representations for the alignments between these ontologies.*

[1]Observe that for every $\mathcal{M}_{ij}$ there is a corresponding $\mathcal{M}_{ji}$ such that $\mathcal{M}_{ij}$ = $\mathcal{M}_{ji}$. Therefore, in the remainder of this paper we will only consider the $\mathcal{M}_{ij}$ where i $<$ j.

Without loss of generality, in this paper we assume that the sets of named concepts for the different ontologies in the network are disjoint.

The domain knowledge of an ontology network is represented by its induced ontology.

Definition 4 *Let $\mathcal{N} = (\mathbb{O}, \mathbb{M})$ be an ontology network, with $\mathbb{O} = \{\mathcal{O}_k\}_{k=1}^n$, $\mathbb{M} = \{\mathcal{M}_{ij}\}_{i,j=1;i<j}^n$. Let $\mathcal{O}_k = (\mathcal{C}_k, \mathcal{I}_k)$. Then the* **induced ontology** *for network $\mathcal{N}$ is the ontology $\mathcal{O}_N = (\mathcal{C}_N, \mathcal{I}_N)$ with $\mathcal{C}_N = \cup_{k=1}^n \mathcal{C}_k$ and $\mathcal{I}_N = \cup_{k=1}^n \mathcal{I}_k \cup_{i,j=1;i<j}^n \mathcal{M}_{ij}$*

2.2. Debugging workfow

For each ontology in the network, the set of candidate missing is-a relations derivable from the ontology network consists of is-a relations between two concepts of the ontology, which can be inferred using logical derivation from the induced ontology of the network, but not from the ontology alone. Similarly, for each pair of ontologies in the network, the set of candidate missing mappings derivable from the ontology network consists of mappings between concepts in the two ontologies, which can be inferred using logical derivation from the induced ontology of the network, but not from the two ontologies and their alignment alone.

Definition 5 *Let $\mathcal{N} = (\mathbb{O}, \mathbb{M})$ be an ontology network, with $\mathbb{O} = \{\mathcal{O}_k\}_{k=1}^n$, $\mathbb{M} = \{\mathcal{M}_{ij}\}_{i,j=1;i<j}^n$ and induced ontology $\mathcal{O}_N = (\mathcal{C}_N, \mathcal{I}_N)$. Let $\mathcal{O}_k = (\mathcal{C}_k, \mathcal{I}_k)$. Then, we define the following.*

(1) $\forall k \in 1..n$: CMI$_k$ = $\{(a, b) \in \mathcal{C}_k \times \mathcal{C}_k \mid \mathcal{O}_N \models a \rightarrow b \wedge \mathcal{O}_k \not\models a \rightarrow b\}$

is the set of candidate missing is-a relations for $\mathcal{O}_k$ derivable from the network.

(2) $\forall i, j \in 1..n, i < j$:

CMM$_{ij}$ = $\{(a, b) \in (\mathcal{C}_i \times \mathcal{C}_j) \cup (\mathcal{C}_j \times \mathcal{C}_i) \mid \mathcal{O}_N \models a \rightarrow b \wedge (\mathcal{C}_i \cup \mathcal{C}_j, \mathcal{I}_i \cup \mathcal{I}_j \cup \mathcal{M}_{ij}) \not\models a \rightarrow b\}$

is the set of candidate missing mappings for ($\mathcal{O}_i, \mathcal{O}_j, \mathcal{M}_{ij}$) derivable from the network.

(3) CMI = $\cup_{k=1}^n$ CMI$_k$ is the set of **candidate missing is-a relations derivable from the network**.

(4) CMM = $\cup_{i,j=1;i<j}^n$ CMM$_{ij}$ is the set of **candidate missing mappings derivable from the network**.

Since the structure of the ontologies and the mappings may contain wrong is-a relations, some of the candidate missing is-a relations and mappings may be derived due to some wrong is-a relations and mappings. Therefore, we need to validate the candidate missing is-a relations for all ontologies and partition them in two sets; $\mathcal{MI}_N$ containing the **missing is-a relations** and $\mathcal{WI}_N$ containing the **wrong is-a relations**. In this case we have that $\mathcal{MI}_N = \cup_{k=1}^n \mathcal{MI}_k$ with $\mathcal{MI}_k$ the set of missing is-a relations in $\mathcal{O}_k$, and $\mathcal{WI}_N = \cup_{k=1}^n \mathcal{WI}_k$ with $\mathcal{WI}_k$ the set of wrong is-a relations in $\mathcal{O}_k$. Similarly, the candidate missing mappings are validated and partitioned into two sets; $\mathcal{MM}_N$ containing the **missing mappings** and $\mathcal{WM}_N$ containing the **wrong mappings**. In this case we have that

$\mathcal{MM}_N = \cup_{i,j;i<j=1}^n \mathcal{MM}_{ij}$ with $\mathcal{MM}_{ij}$ the set of missing mappings between $\mathcal{O}_i$ and $\mathcal{O}_j$, and
$\mathcal{WM}_N = \cup_{i,j;i<j=1}^n \mathcal{WM}_{ij}$ with $\mathcal{WM}_{ij}$ the set of wrong mappings between $\mathcal{O}_i$ and $\mathcal{O}_j$.

Definition 6 *Let $\mathcal{N} = (\mathbb{O}, \mathbb{M})$ be an ontology network, with $\mathbb{O} = \{\mathcal{O}_k\}_{k=1}^n$, $\mathbb{M} = \{\mathcal{M}_{ij}\}_{i,j=1;i<j}^n$ and induced ontology $\mathcal{O}_N = (\mathcal{C}_N, \mathcal{I}_N)$. Let $\mathcal{O}_k = (\mathcal{C}_k, \mathcal{I}_k)$. Let $\mathcal{MI}_k$ and $\mathcal{WI}_k$ be the missing, respectively wrong, is-a relations for ontology $\mathcal{O}_k$ and let $\mathcal{MI}_N = \cup_{k=1}^n \mathcal{MI}_k$ and $\mathcal{WI}_N = \cup_{k=1}^n \mathcal{WI}_k$. Let $\mathcal{MM}_{ij}$ and $\mathcal{WM}_{ij}$ be the missing, respectively wrong, mappings between ontologies $\mathcal{O}_i$ and $\mathcal{O}_j$ and let $\mathcal{MM}_N = \cup_{i,j=1;i<j}^n \mathcal{MM}_{ij}$ and $\mathcal{WM}_N = \cup_{i,j=1;i<j}^n \mathcal{WM}_{ij}$. A* **structural repair for** $\mathcal{N}$ **with respect to** $(\mathcal{MI}_N, \mathcal{WI}_N, \mathcal{MM}_N, \mathcal{WM}_N)$, *denoted by $(\mathcal{R}^+, \mathcal{R}^-)$, is a pair of sets of is-a relations and mappings, such that*

(1) $\mathcal{R}^- \cap \mathcal{R}^+ = \emptyset$

(2) $\mathcal{R}^- = \mathcal{R}_M^- \cup \mathcal{R}_I^-$; $\mathcal{R}_M^- \subseteq \cup_{i,j=1,i<j}^n \mathcal{M}_{ij}$; $\mathcal{R}_I^- \subseteq \cup_{k=1}^n \mathcal{I}_k$

(3) $\mathcal{R}^+ = \mathcal{R}_M^+ \cup \mathcal{R}_I^+$; $\mathcal{R}_M^+ \subseteq \cup_{i,j=1,i<j}^n ((\mathcal{C}_i \times \mathcal{C}_j) \setminus \mathcal{M}_{ij})$; $\mathcal{R}_I^+ \subseteq \cup_{k=1}^n ((\mathcal{C}_k \times \mathcal{C}_k) \setminus \mathcal{I}_k)$

(4) $\forall k \in 1..n : \forall (a,b) \in \mathcal{MI}_k$: $(\mathcal{C}_k, (\mathcal{I}_k \cup (\mathcal{R}_I^+ \cap (\mathcal{C}_k \times \mathcal{C}_k))) \setminus \mathcal{R}_I^-) \models a \rightarrow b$

(5) $\forall i,j \in 1..n, i<j : \forall (a,b) \in \mathcal{MM}_{ij}$:
$((\mathcal{C}_i \cup \mathcal{C}_j), (\mathcal{I}_i \cup ((\mathcal{C}_i \times \mathcal{C}_i) \cap \mathcal{R}_I^+) \cup \mathcal{I}_j \cup ((\mathcal{C}_j \times \mathcal{C}_j) \cap \mathcal{R}_I^+) \cup \mathcal{M}_{ij} \cup ((\mathcal{C}_i \times \mathcal{C}_j) \cap \mathcal{R}_M^+)) \setminus \mathcal{R}^-) \models a \rightarrow b$

(6) $\forall (a,b) \in \mathcal{WI}_N \cup \mathcal{WM}_N \cup \mathcal{R}^-$: $(\mathcal{C}_N, (\mathcal{I}_N \cup \mathcal{R}^+) \setminus \mathcal{R}^-) \not\models a \rightarrow b$

The definition states that (1) is-a relations and mappings cannot be added and removed at the same time, (2) the removed mappings come from the original alignments and the removed is-a relations come from the original asserted is-a relations in the ontologies, (3) the added mappings were not in the original alignments and the added is-a relations were not original is-a relations in the ontologies, (4) every missing is-a relation is derivable from its repaired host ontology, (5) every missing mapping is derivable from the repaired host ontologies of the mapped concepts and their repaired alignment, and (6) no wrong mapping, wrong is-a relation or removed mapping or is-a relation is derivable from the repaired network.

Definition 7 *Let (x_1, y_1) and (x_2, y_2) be two different is-a relations in the same ontology $\mathcal{O}$ (i.e., $x_1 \not\equiv x_2$ or $y_1 \not\equiv y_2$), then we say that (x_1, y_1) is* **more informative than** (x_2, y_2) *iff $\mathcal{O} \models x_2 \rightarrow x_1 \wedge y_1 \rightarrow y_2$.*

It follows that if (x_1, y_1) is more informative than (x_2, y_2) and $\mathcal{O} \models x_1 \rightarrow y_1$ then $\mathcal{O} \models x_2 \rightarrow y_2$. Therefore, adding or removing more informative repairing actions, adds or removes more knowledge than less informative repairing actions.

2.3 Algorithms

2.3.1 Detecting and validating candidate missing is-a relations and mappings

In the restricted setting where we assume that all existing is-a relations in the ontologies and all existing mappings in the alignments are correct (and thus the debugging problem does not need to consider wrong is-a relations and mappings), it can be shown that all candidate missing is-a relations and mappings[2] will be repaired when we repair the candidate missing is-a relations and mappings between *mapped concepts*.

Theorem 1 *Let* $\mathcal{N} = (\mathbb{O}, \mathbb{M})$ *be an ontology network with* $\mathbb{O} = \{\mathcal{O}_k\}_{k=1}^n$ *the set of the ontologies in the network and* $\mathbb{M} = \{\mathcal{M}_{ij}\}_{i,j=1;i<j}^n$ *the set of representations for the alignments between these ontologies. Further, assume that all is-a relations in the ontologies and all mappings in the alignments are correct. Then the following holds:*
(i) For each candidate missing is-a relation (a, b) *in ontology* $\mathcal{O}_i$*, there exists a candidate missing is-a relation* (x, y) *in ontology* $\mathcal{O}_i$ *where* x *and* y *are mapped concepts in alignments between* $\mathcal{O}_i$ *and other ontologies in the network, such that the repairing of* (x, y) *also repairs* (a, b)*.*
(ii) For each candidate missing mapping (c, d) *such that* $c \in \mathcal{O}_i$ *and* $d \in \mathcal{O}_j$ *with* $i \neq j$*, there exists a candidate missing mapping* (x, y) *such that* $x \in \mathcal{O}_i$ *and* $y \in \mathcal{O}_j$*,* x *is a mapped concept in an alignment between* $\mathcal{O}_i$ *and another ontology in the network and* y *is a mapped concept in an alignment between* $\mathcal{O}_j$ *and another ontology in the network, such that the repairing of* (x, y) *also repairs* (c, d)*.*

Proof. Assume (a, b) is a candidate missing is-a relation in $\mathcal{O}_i$. According to the definition of candidate missing is-a relation, the relation $a \rightarrow b$ is not derivable from $\mathcal{O}_i$ but derivable from the ontology network. So, there must exist at least one concept from another ontology in the network, for instance z, such that $\mathcal{O}_N \models a \rightarrow z \rightarrow b$. Because concepts a and z reside in different ontologies, the relation $a \rightarrow z$ must be supported by a mapping between a concept x in $\mathcal{O}_i$ and a concept x' in another ontology, e.g. $\mathcal{O}_r$, in the network, such that $(x,x') \in M_{ir}$ (if i < r) or $(x,x') \in M_{ri}$ (if r < i), and $\mathcal{O}_N \models a \rightarrow x \rightarrow x' \rightarrow z$. Likewise, for concepts z and b, the relation $z \rightarrow b$ must also be supported by a mapping between a concept y in $\mathcal{O}_i$ and a concept y' in another ontology, e.g. $\mathcal{O}_s$, in the network, such that $(y',y) \in M_{sj}$ (if s < j) or $(y',y) \in M_{js}$ (if j < s), such that $\mathcal{O}_N \models z \rightarrow y' \rightarrow y \rightarrow b$. We can then deduce that $x \rightarrow y$ is derivable from the ontology network because $\mathcal{O}_N \models a \rightarrow x \rightarrow x' \rightarrow z \rightarrow y' \rightarrow y \rightarrow b$. Since $a \rightarrow b$ is not inferrable from $\mathcal{O}_i$, the relation $x \rightarrow y$ can not be inferred from $\mathcal{O}_i$ either. This means that (x, y) is also a candidate missing is-a relation in $\mathcal{O}_i$, and the repairing of (x, y) also repairs (a, b). This proves statement (i). A similar proof can be given for statement (ii).

♣

[2]In this setting all candidate missing is-a relations are also missing is-a relations, and all candidate missing mappings are also missing mappings.

In the algorithms we use the notion of knowledge base. The notion that we define here is a restricted[3] variant of the notion as defined in description logics [1].

Definition 8 *Let $\mathcal{C}$ be a set of named concepts. A* **knowledge base** *is then a set of axioms of the form $A \rightarrow B$ with $A \in \mathcal{C}$ and $B \in \mathcal{C}$. A model of the knowledge base satisfies all axioms of the knowledge base.*

In the algoritms we initialize knowledge bases with an ontology. This means that for ontology $\mathcal{O}$=$(\mathcal{C},\mathcal{I})$ we create a knowledge base such that (A,B) $\in \mathcal{I}$ iff $A \rightarrow B$ is an axiom in the knowledge base.
For the knowledge bases, we assume that they are able to do deductive logical inference. Further, we need the following reasoning services. For a given statement the knowledge base should be able to answer whether the statement is entailed by the knowledge base.[4] If a statement is entailed by the knowledge base, it should be able to return the derivation paths (explanations) for that statement. For a given named concept, the knowledge base should return the super-concepts and the sub-concepts.
The knowledge bases can be implemented in several ways. For instance, any description logic system could be used. In our settting where we deal with taxonomies we have used an efficient graph-based implementation. We have represented the ontologies using graphs where the nodes are concepts and the directed edges represent the is-a relation. The entailment of statements of the form $a \rightarrow b$ can be checked by transitively following edges starting at a. If b is reached, then the statement is entailed, otherwise not. If $a \rightarrow b$ is entailed, then the derivation paths are all the different paths obtained by following directed edges that start at a and end at b. The super-concepts of a are all the concepts that can be reached by following directed edges starting at a. The sub-concepts of a are all the concepts for which there is a path of directed edges starting at the concept and ending in a.

2.3.3 Repairing wrong is-a relations and mappings

Definition 9 *(similar definition as in [2]) Given an ontology $\mathcal{O} = (\mathcal{C},\mathcal{I})$, and $(a,b) \in \mathcal{C} \times \mathcal{C}$ an is-a relation derivable from $\mathcal{O}$, then, $\mathcal{I}' \subseteq \mathcal{I}$ is a* **justification** *for (a,b) in $\mathcal{O}$, denoted by $\mathsf{Just}(\mathcal{I}', a, b, \mathcal{O})$ iff (i) $(\mathcal{C},\mathcal{I}') \models a \rightarrow b$; and (ii) there is no $\mathcal{I}'' \subsetneq \mathcal{I}'$ such that $(\mathcal{C},\mathcal{I}'') \models a \rightarrow b$. We use $\mathsf{All\_Just}(a, b, \mathcal{O})$ to denote the set of all justifications for (a,b) in $\mathcal{O}$.*

To compute the justifications for $a \rightarrow b$ in our graph-based implementation, all the different paths obtained by following directed edges that start at a and end at b are collected. Among these the minimal ones (w.r.t $\subseteq$) are retained.

[3]We use only concept names and no roles. The axioms in the TBox are of the form $A \dot{\sqsubseteq} B$ or $A \doteq C$, and the ABox is empty.
[4]In our setting, entailment by ontology can be reformulated as entailment by knowledge base.

References

1. Baader F, Calvanese D, McGuinness D, Nardi D, Patel-Schneider P: *The description logic handbook*. Cambridge University Press 2003.

2. Jimenez-Ruiz E, Grau BC, Horrocks I, Berlanga R: **Ontology Integration Using Mappings: Towards Getting the Right Logical Consequences**. In *Proceedings of the 6th European Semantic Web Conference*, *Volume 5554 of* LNCS, Springer 2009:173–187.
